# Supplementary material for: Are carbon emissions trading and green financial instruments synergistic? -Comprehensive quantitative research based on content analysis
Source: PLoS One. 2024 Mar 7;19(3):e0298601. doi: 10.1371/journal.pone.0298601 (PMC10919601; doi:10.1371/journal.pone.0298601)
Supplement: S1 Data — (ZIP) [file pone.0298601.s001.zip › Data/地级市Z_F工作报告.zip!/░▓╗╒/╦▐╓▌╩╨/└┤╫╘╣1⁄2╓┌║┼í╛┬φ┐╦╩2╛▌═°í┐_2012.docx]

刚刚过去的一年，全市上下在市委的坚强领导下，认真贯彻党的十七大和十七届三中、四中、五中、六中全会以及省第九次党代会、市第四次党代会精神，全面落实科学发展观，主动作为，苦干实干，圆满完成市三届人大三次会议确定的各项任务，实现“十二五”良好开局。

预计全市地区生产总值780亿元，增长13.8%，年增量首超百亿；财政收入64.1亿元，增长48.5%，连跨两个十亿元台阶，增速居全省第一；固定资产投资460亿元，增长30%以上；社会消费品零售总额230亿元，增长18%;城镇居民人均可支配收入17160元，农民人均纯收入5580元，均增长17%以上，主要经济指标增速进入全省前八位。

一年来，我们主要做了以下工作：

一、聚焦工业扩张，发展方式实现新转变。全面实施“7233”工程，全市规模以上工业增加值280亿元，增长25%，增速保持全省领先；工业投资250亿元，增长40%。积极壮大市场主体，新增产值超亿元企业100户。工业经济效益综合指数306%，创历史新高。加速培育新兴产业，首文软磁、维多甜味剂、格微光电、万兴科技完成一期建设，诺业生物、湛蓝光电、品青果糖、大诚明科技等项目开工建设。全力打好节能减排攻坚战，单位生产总值能耗和主要污染物排放达到省考核目标。

二、狠抓投资消费，经济发展增添新动力。全年累计向国家和省争取资金15亿元；列入省“861”项目完成投资130亿元，增长89%;深入谋划“5161”工程，项目储备规模超万亿。一批事关全局的重大项目取得突破。永通电缆、光大生物质能发电、合成革一期、皖神面粉、锦龙制衣等项目建成投产，国电热电联产、祁南二矿、海螺水泥、冠军陶瓷等项目加快推进。京沪高铁正式通车，宿淮铁路、徐明高速、泗许高速二期、济祁高速、山闵路进展顺利，农村公路建成通车290公里。首座110千伏智能变电站建成投运。

三、夯实“三农”基础，县域经济激发新活力。战胜60年一遇的冬春连旱，粮食总产突破80亿斤，实现八连增。加快农业产业化转型升级，农产品加工产值550亿元，市级以上龙头企业225家，年营销收入330亿元，发展标准化养殖小区73个、农民专业合作组织900个，农业产业化工作保持全省先进，带动农民户均增收2680元。现代农业示范区扎实推进，农田水利三年恢复性工程顺利完成。修建通村公路119公里，解决23万农村人口饮水安全问题。林木产业、农机化综合水平继续保持全省领先。全国农村改革试验区正式获批。完成土地流转面积55万亩，土地整治5万亩。全面提速县域经济发展。

四、加速城乡建设，城乡面貌呈现新变化。城乡规划管理持续加强，新一轮城市总规划上报省政府待批，灵璧、砀山、萧县总规划通过评审，道东片区、汴北物流园等控详规划完成编制。主城区以“1133”工程为龙头，投资超百亿，实施200项重点工程，新建续建道路40公里。“五化工程”有效实施，改造绿地10万平方米，完成亮化工程64个，全面完成出租车更新，城市品位进一步提升。征收房屋和控违220万平方米，建设安置房1.5万套。突出抓好县城建设，发展框架逐步拉开，城市功能不断完善。

五、承接产业转移，改革开放取得新成果。持续给力招商引资，全年实际到位内资466亿元，增长55%;实际利用外资2.3亿美元，增长57%。深入开展皖粤经贸合作，签约项目总数及投资额居全省前列。成功举办第六届黄淮海地区农博会、砀山果蔬论坛。市经济开发区扩区面积10平方公里，“一区六园”建设框架逐步拉开，鞋业基地21个项目开工建设，化工园区20个项目签署入园协议。宿马经济区总规通过评审，起步区建设与招商同步展开。高新技术产业园入驻智慧云计算等6家企业。5个县区开发区实现工业总产值140亿元，128个项目开工投产。14个市级乡村工业园入驻企业198家。深化重点领域改革。鼓励促进民间投资，全年新增私营企业2413家，个体工商户1.7万户。

六、壮大文化实力，文化建设彰显新亮点。大力实施文化惠民工程，市博物馆接待观众10万余人次，广播电视由村村通向户户通延伸，建成乡镇综合文化站39个，农家书屋在全省率先实现全覆盖；全年送戏下基层进社区500余场次、送电影3万多场。不断壮大文化产业，宿州文化产业园、神游世界动漫产业园等项目相继落户，文化产业增加值超10亿元。实施文艺精品工程。

七、保障改善民生，幸福指数得到新提升。加大33项民生工程推进力度，投入资金42.6亿元，增长56%，实施工作跻身全省先进。9件惠民实事如期完成。实施积极的就业政策，新增城镇就业5.2万人，农民工技能培训4.2万人次，城镇登记失业率控制在4.3%。社会保障体系不断完善，城乡居民社会养老保险试点顺利推进。加强薄弱学校建设，免除学杂费4.4亿元，完成151所学校标准化建设，校舍安全加固改造97.2万平方米。强化公共卫生服务，新农合参合率达97.6%，重大疫病预防控制有效落实，社区、乡村卫生服务网络逐步完善。住房保障工作位列全省先进。

八、创新社会管理，和谐宿州开创新局面。不断创新社会管理模式，城乡社区自治和服务功能进一步增强。扎实推进“平安宿州”建设，健全社会治安防控体系，人民群众安全感稳步上升。信访秩序持续好转。“六五普法”启动实施。国防动员、人民防空和双拥优抚工作取得新成绩，争创全国双拥模范城顺利通过考核验收。低生育水平持续稳定，出生人口性别比偏高势头得到遏制。

在推进经济社会发展的同时，我们高度重视政府自身建设，努力塑造负责任、敢担当、干实事、有作为的政府形象。注重听取各方意见和建议，自觉接受人大法律监督、政协民主监督和社会舆论监督，按期办结人大代表议案3件、建议77件、政协委员提案246件。深化行政审批制度改革，审批效率大幅提升。深化政务公开，畅通政风行风热线，73家部门设立网络发言人。建立健全行政决策机制，廉政建设取得新成效。

二、2012年工作总体安排

2012年，是深入实施“十二五”规划的关键之年。在这个重要关口，我们必须树立担当意识，坚定必胜信念，锐意进取，争先进位，才能在更高起点上实现新的跨越。

今年工作的总体要求是：高举中国特色社会主义理论伟大旗帜，以邓小平理论和“三个代表”重要思想为指导，深入贯彻落实科学发展观，按照市第四次党代会的部署，坚定不移地推进工业扩张、农业提升、城镇扩容三大战略，以开展“园区和城乡建设突破年”、“社会管理创新年”活动为抓手，抢抓新一轮产业转移的重大机遇，着力在承接产业转移中扩张工业总量，着力在推进产城一体中加速城镇化步伐，着力在促进三化同进中发展现代农业，着力在加强社会管理创新中促进社会和谐，努力实现经济发展稳中求进、好中求快、又好又快，力争走在皖北地区崛起前列，为加速建成四省交汇的区域中心城市奠定坚实基础。

今年经济社会发展的主要目标是：全市地区生产总值增长13%左右，财政收入增长15%以上，固定资产投资增长25%以上，社会消费品零售总额增长16.5%以上，城乡居民收入增长15%以上，城镇登记失业率控制在4.5%以内，人口出生率控制在13.5‰以内，单位生产总值能耗和化学需氧量、氨氮、二氧化硫、氮氧化物排放量达到省控目标。

（一）全力扩张工业总量

围绕工业五年扩张，深入实施“7233”工程，确保工业固定资产投资增长30%以上，规模以上工业增加值增长22%以上。

着力做大主导产业。坚持以大项目带动大产业，以“5161”工程为抓手，全年组织实施亿元以上项目300个，其中10亿元以上项目40个。完善项目推进机制，加大项目考核权重，确保国电热电联产、海螺水泥、龙王庙煤矿、中元复合肥、恒顺工贸、西地亚板材等30个亿元以上项目建成投产，加快永通林浆纸一体化、金黄庄煤矿、合成革二期、诸暨袜业产业园、纺织服装产业城等200个亿元以上项目建设。大力实施重点产业规划，积极发展纺织鞋服首位产业，改造提升煤电能源、化工建材、板材家具、食品加工、装备制造等主导产业。

着力做强现有企业。坚持抓大扶小、促强带弱，完善企业帮扶机制，推动小微企业上规模、规模企业上台阶。加快自主创新步伐，新建两家省级研发中心。抓好30户上市后备企业培育，确保皖北药业上市。加强工业运行调度，切实保障要素供给。

着力抓好节能减排。严格执行节能减排责任制和“一票否决制”。全面实行新上项目节能评估和审查制度，推进8个节能项目建设。继续抓好污染防治和生态建设，严格新建项目环评和“三同时”制度，加强饮用水源地保护。

（二）全面抓好“三农”工作

始终抓紧抓牢“三农”工作，夯实农业农村发展基础，力争粮食总产达到81亿斤，农民增收800元以上。

大力发展现代农业。深入实施“4168”工程，主攻四大支柱产业，做大做强粮食、果蔬、畜禽、板材等省市级产业化示范区。持续推动小麦高产攻关、玉米振兴计划，支持县区争创小麦玉米单产千斤县。实施畜牧业升级计划、蔬菜产业提升行动。加快60万亩现代农业核心示范区建设。全面实施农业产业化“181”转型倍增计划，力争农产品加工总产值突破600亿元，市级以上龙头企业达到280家。加强农产品质量安全体系建设，全力招引国内外知名龙头大企业。启动实施国家农村改革试验区工作，创新现代农业经营组织体系。

拓宽农民增收渠道。围绕农民增收五年倍增目标，着力构建多元化农民增收支撑体系。大力开展农村劳动力转移培训，增加农民工资性收入。依法推动土地流转和适度规模经营，增加农民财产性收入。

改善农村发展环境。启动实施农田水利三年提升规划，新建高标准农田12.1万亩，新增节水灌溉面积7万亩。全力推进新汴河综合治理工程，完成5条中小河流治理，除险加固两座病险水库，解决23万人饮水安全问题。

（三）加速推进城镇扩容

按照城乡一体、区域联动发展的理念，全面掀起园区和城乡建设突破年活动，投入180亿元，实施五大类277个重点项目，建成道路120公里、征收房屋220万平方米、新增绿地200万平方米。

做大做强中心城市。坚持东向高铁、北跨汴河、南展新城、西越高速，加快构筑“132”格局，建设宜居宜业宜游城市。突出规划引领，新编完善4个分区规划、21个专项规划、10个园区规划、8个重点街区规划。加快五大片区建设，实施大外环、高铁快速通道工程，拉开城市框架。以创建国家园林城市和省文明城市为目标，强化区、街道和社区城市管理责任，推进土地征用、房屋征收、控违拆违重心下移，整顿交通秩序，强化渣土管理，实施“五化”工程，创新“公众城管”模式，完善城市管理体制，提升城市管理水平。

加快县城和小城镇建设。搞好县、镇、村三级规划衔接，加速推进四个县城建设，加大资金投入，完善基础设施，提升功能档次。

加强基础设施建设。加快推进宿淮铁路、徐明高速、泗许高速二期、济祁高速、山闵路建设，力促郑徐客运专线开工，启动206国道改线工程，改造加固农村公路危桥120座，改造升级县乡公路200公里，加速构建现代道路交通体系。

（四）主动承接产业转移

以改革增活力，以开放促发展，不断拓宽改革的深度和开放的力度，进一步提升承接产业转移的能力和水平。

积极推进各类改革。放宽准入条件，发展非公经济，完善促进民间投资体制，力争新增民营企业2900户。深化财政预算管理体制改革，规范政府采购行为。继续抓好国有集体企业改革。规范土地管理，加强节约集约用地，依法清理闲置土地。加快事业单位人事制度和收入分配制度改革。

全力主攻招商引资。坚持以税收和就业实绩检验招商实效，持续保持招商引资强劲势头，大规模、快节奏、高频率推进招商引资。注重招大引强、招强引链、挑商选资。创新招商方式，巩固皖粤对接成果，密切跟踪服务，狠抓项目调度，全面提高合同履约率、资金到位率和项目开工率。

聚力打造园区平台。围绕“思想解放先导区、特色产业示范区、优质要素富集区、幸福生活新城区”的定位，加速各类园区建设，推动产城一体、功能集聚、扩容升级。

（五）着力发展第三产业

加大服务业投入，促进服务业比重提高、结构优化、竞争力提升，力争服务业增加值增长12%以上。

壮大提升现代服务业。坚持外引与内培并举、生产性与生活性并重，着力打造以宿城为中心、四个县城为次中心的区域性物流中心。落实加快发展服务业若干政策，改造提升商贸物流、邮政快递等传统服务业，扶持培育服务外包、会展、动漫等新兴服务业。

突出发展旅游产业。制定实施旅游业三年行动计划，完善规划、打牢基础、强化措施、整体推进。加快闵子祠、梅村生态园、皖东北革命纪念馆等重点旅游景点建设，推进高档星级酒店建设，加大旅游产品开发力度，力争有新突破。

持续推动金融创新。积极引进交通银行等股份制商业银行在我市设立分支机构，加快村镇银行建设，扶持徽商银行、淮海银行县域机构全覆盖，推进农村信用社改革组建农商行。着力破解中小微企业融资难题，申报发行中小企业集合债券。

（六）大力振兴县域经济

振兴县域经济事关兴宿富民大业，必须持之以恒加大支持力度，激发内生动力，使县域经济成为我市加速崛起的重要支撑。

推动县域经济晋位升级。把工业突破作为助推县域经济的重要引擎，突出抓好一批百亿元产业集群，以特色产业带动县域三化同进。

加大政策扶持力度。积极落实支持皖北发展“双十条”政策，加大对县区项目、用地等扶持力度。积极争取点供用地，大力推进农村土地整治、增减挂钩工作，增加建设用地指标。完善县域经济考核措施，积极推进扩权强镇，催生一批工业强镇、商贸重镇、旅游名镇。

（七）推进文化大发展

文化是人民的精神家园，必须不断增强文化自觉和文化自信，以更大的力度推进文化改革发展，进一步提升文化软实力。

大力建设和谐文化。扎实推进社会主义核心价值体系建设，精心组织全国道德模范推荐、“身边好人”评选活动，切实加强社会公德、职业道德、家庭美德和个人品德教育，全面提升公民思想道德素质。继续开展省级文明城市、文明县城、文明村镇和市级文明单位创建工作。

繁荣发展文化事业。大力推进文化惠民工程，完善城乡公共文化服务体系。实施文化保护工程，做好24个国家、省级非物质文化遗产的保护工作。实施文化精品工程，鼓励文学艺术创作。实施文化下乡工程。

加速培育文化产业。加快神游世界动漫产业园、宿州文化产业园、钟馗文化园、天鹅洲文化园、星光影视基地等项目建设。加大政策扶持力度，做大做强灵璧奇石、萧县书画、泗县戏曲、砀山唢呐、埇桥马戏五大文化产业，提升文化产业整体实力和竞争力。

（八）统筹发展社会事业

民生连着民心，民心决定民意，要竭尽全力把保障改善民生的各项措施落到实处，努力让全市人民生活得更加幸福、更有尊严。

完善就业和社会保障体系。高度重视就业难、招工难矛盾，加强供需衔接，促进高校毕业生、农村富余劳动力等重点人群就业。坚持以创业带动就业，争创省级创业型城市。加大社会保险扩面征缴力度，完善社会救助体系。加大医疗救助力度，健全新农合机制，确保参合率达95%以上。实施居民收入倍增规划，不断提高城乡居民生活水平。

全面发展各项社会事业。坚持教育优先，规范中小学办学行为。继续实施学前教育三年行动计划，新建、改扩建幼儿园39所。加快教育结构调整，推进151所标准化学校建设，加固改造中小学校舍19.4万平方米。完善城乡义务教育经费保障机制，积极解决进城务工人员子女入学和农村留守儿童问题。启动教育园区建设。积极发展卫生事业。加快推进县级以下卫生服务机构规范化建设。实施全民健身计划，抓好街头、社区等公共场所体育设施配套建设。加强流动人口管理，抓好出生人口性别比治理，稳定低生育水平。

切实加强和创新社会管理。依法整顿规范市场秩序，强化产品质量、食品药品安全监管，打击商业欺诈和非法传销。加强价格监管，保持市场价格基本稳定。全面推进和谐社区建设，强化城乡社区自治和服务功能。推进企业工资集体协商制度，构建和谐劳动关系。依法管理民族宗教事务。支持工会、共青团、妇联参与社会管理。继续抓好超限超载治理。落实安全生产责任，严防重特大事故发生。加强政府应急管理，提高防震减灾、保障公共安全和处置突发事件的能力。加强国防动员、人防和民兵预备役建设，争创双拥模范城，促进军民融合发展。深化书记带头大走访活动成果。完善矛盾纠纷排查化解机制。深入推进“平安宿州”建设。

今年，我们在深入推进33项民生工程的基础上，本着“普惠、解难、量力、当年”的原则，再办10件惠民实事，让更多的群众享受改革发展成果。

1、打通城市“断头路”12条（含县城）。

2、全市完成10个“四有社区”建设。

3、实施清水工程，完成20公里河道综合治理。

4、完成农村危房改造4000户，村庄整治50个。

5、全市新建各类保障性住房2.3万套（间），改造道东棚户区30万平方米。

6、实施城乡净化工程，建成垃圾处理场5个、垃圾中转站45个。

7、全市兴建26处公园广场游园，其中市区7处。

8、继续实施市区供热工程，铺设主管网5.2公里，1.2万户居民用上暖气。

9、继续推进供水管网改造，自来水一户一表改造5000户。

10、完成马山公墓道路修复及环境整治。

三、打造人民满意的服务型政府

加强政府自身建设，是百姓之期望、履职之必须。要把对人民负责作为最高准则，深入开展“机关效能建设年”活动，把提速提效贯穿于政府各项工作之中，强化执行力，保持快节奏，努力建设人民满意的服务型政府。

真抓实干，高效作为，提振干事精神。坚持效能为先，倡导勇于担当、雷厉风行的工作作风，建立高效快捷、迅速果断的执行机制，形成奖优罚劣、失责必问的工作导向。强化目标管理，健全考核督查，严格行政问责。政府班子率先垂范，对关键环节和重点难点问题一抓到底。坚决摆脱文山会海，严格控制各类检查评比，集中精力、群策群力谋大事、促发展。

转变职能，高效服务，恪守为民之本。大力弘扬以实干立身的政绩观，切实转变政府职能。继续深化“两集中、两到位”改革，清理下放行政审批事项，实行网上审批市县区联动，深入推进“四个一”工程。始终保持“一枝一叶总关情”的公仆情怀，站稳群众立场，开展走访活动，满腔热情为群众办实事、解难事。

阳光规范，高效执政，务求履职尽责。把依法行政贯穿于政府决策、执行、监督全过程，切实规范行政行为。推进政务公开，深化网络问政，做到决策部署充分反映民意、广泛集中民智、切实珍惜民力，推进行政决策科学化、民主化。更加自觉地接受人大、政协、社会公众和新闻舆论监督，凝聚发展智慧，形成攻坚合力。

勤勉敬业，高效廉洁，永葆公仆本色。面对人民的期盼，全体政府公务人员要堂堂正正做人、踏踏实实干事，夙兴夜寐，永不懈怠。严格遵守廉洁自律各项规定，加强惩治和预防腐败体系建设。努力塑造风清气正、勤勉尽责的政府形象。
